# Mechanisms underlying cognitive impairment and management strategies in type 2 diabetes

**DOI:** 10.3389/fendo.2025.1655768

**Published:** 2025-10-15

**Authors:** Xiaolin Chen, Yan Huang, Xiaoxing Xiong

**Affiliations:** ^1^ Department of Endocrinology, Renmin Hospital of Wuhan University, Wuhan, Hubei, China; ^2^ Department of Neurosurgery, Renmin Hospital of Wuhan University, Wuhan, Hubei, China

**Keywords:** type 2 diabetes, cognitive impairment, insulin resistance, neuroinflammation, glycemic control, neuroprotection

## Abstract

Type 2 diabetes (T2D) is increasingly recognized as a risk factor for cognitive impairment, ranging from mild cognitive impairment (MCI) to dementia. The underlying mechanisms involve a complex interplay of hyperglycemia, insulin resistance, neuroinflammation, oxidative stress, vascular dysfunction, and amyloid pathology. Effective management strategies remain an area of active investigation. This review explores the pathophysiological mechanisms linking T2D to cognitive dysfunction and evaluates current and emerging therapeutic strategies to preserve cognitive function in diabetic patients. Chronic hyperglycemia and insulin resistance impair neuronal function and synaptic plasticity, while microvascular complications contribute to cerebral hypoperfusion and white matter lesions. Additionally, metabolic disturbances exacerbate neurodegenerative processes, further compromising cognitive health. Effective management strategies for cognitive impairment in T2D include regular cognitive screening, stringent glycemic control, lifestyle modifications, comprehensive cardiovascular risk management, patient education and pharmacological interventions such as metformin, GLP-1 receptor agonists (GLP1RAs), and sodium-glucose cotransporter 2 (SGLT2) inhibitors, which may offer neuroprotective benefits. In this review, we conclude that cognitive impairment in T2D results from complex, interrelated mechanisms requiring early intervention and personalized strategies. While current therapies focus on metabolic and vascular risk reduction, future research should prioritize biomarker discovery, mechanism-driven treatments, and long-term clinical trials to optimize outcomes. A proactive, integrated care model is essential to mitigate cognitive decline in this high-risk population.

## 1 Introduction

A growing body of literature has demonstrated that type 2 diabetes (T2D) is increasingly recognized as a significant risk factor for cognitive impairment, including mild cognitive impairment (MCI), dementia, and Alzheimer’s disease (AD), particularly in domains of memory, executive function, and processing speed (1, 2). All cognitive impairment could lead to reduced treatment compliance, medication management, and self-care ability in T2D patients. Cognitive impairment in T2D manifests through a characteristic pattern of deficits that primarily affect memory, executive function, and processing speed (3). Patients often experience gradual declines in episodic memory, struggling to recall recent events or retain new information, alongside noticeable difficulties in complex tasks requiring planning, decision-making, and mental flexibility (4). Executive dysfunction is particularly prominent, leading to impaired problem-solving abilities and reduced capacity to multitask (5, 6). Additionally, slowed information processing results in delayed responses during cognitive tasks and conversations (7). Many patients also report increased distractibility and working memory challenges, while visuospatial difficulties may emerge in later stages (8). These cognitive changes frequently co-occur with mood disturbances, such as depression, and often correlate with longer diabetes duration, poor glycemic control, and the presence of microvascular complications (9). Hence, early recognition of these manifestations is critical for timely intervention to preserve cognitive function in diabetic patients (10).

The growing prevalence of T2D worldwide is expected to contribute to a significant increase in dementia cases (9). Cognitive impairment in T2D patients not only diminishes personal autonomy—increasing reliance on caregivers for daily activities—but also exacerbates socioeconomic burdens through lost productivity and higher medical costs associated with dementia care. Early intervention can preserve functional abilities, allowing individuals to maintain meaningful social roles, employment, and community engagement for longer periods. Furthermore, mitigating cognitive decline reduces caregiver strain, which disproportionately affects families and healthcare systems in aging populations. Public health initiatives targeting diabetes-related cognitive risks could yield substantial societal benefits by delaying disability onset, reducing long-term care needs, and promoting healthier aging. Given the rising global prevalence of T2D, addressing its cognitive consequences is not just a medical imperative but a societal necessity to foster resilient communities and sustainable healthcare systems for future generations.

Chronic hyperglycemia, insulin resistance, and microvascular dysfunction drive pathological processes such as neuroinflammation, oxidative stress, and amyloid deposition, which collectively impair synaptic plasticity and cognitive function (4). While glycemic control remains a cornerstone of diabetes management, certain antidiabetic agents may offer additional neuroprotective benefits beyond glucose-lowering effects (11). Sodium-glucose cotransporter 2 (SGLT2) inhibitors, a newer class of glucose-lowering drugs, have emerged as promising candidates for mitigating diabetes-associated cognitive decline (12, 13). Beyond their renal and cardiovascular benefits, preclinical and clinical evidence suggests that SGLT2 inhibitors may improve cognitive outcomes through multifaceted mechanisms, including enhanced cerebral metabolism, reduced neuroinflammation, improved endothelial function, and direct neuroprotective effects (14). This review explores the pathophysiological links between T2D and cognitive impairment, examines the potential mechanisms by which early intervention and personalized strategies confer cognitive benefits, and discusses the clinical implications of these findings for diabetes management and dementia prevention.

## 2 Evidence of cognitive impairment associated with T2D

Cognitive impairment is a well-documented complication of T2D, affecting memory, executive function, attention, and processing speed (15). Additionally, the risk of developing Alzheimer’s disease and other forms of dementia is approximately twice as high in individuals with T2D (16). Moreover, the rate of progression from MCI to dementia is 1.5 to 3.0 times higher in patients with T2D than in those without T2D (17). The Rotterdam Study and the Framingham Heart Study have demonstrated that T2D is associated with a 50-100% increased risk of dementia (18, 19). The Health and Retirement Study provided evidence that individuals with T2D had a 1.66-fold higher likelihood of cognitive impairment without dementia, as compared to those with normal cognition, among participants of European ancestry (20). In the rural China, the prevalence of MCI in older individuals with T2D was 53.48% (21). A systematic evaluation showed that incidence of cognitive impairment in elderly patients with T2D in China was 48%, with a higher incidence in population who were female, with a lower education level, a low income, no spouse, and living alone (22). A meta-analysis of T2D cases twelve years ago reported an increase of 73% in the risk of all types of cognitive impairment and 56% in the risk of AD in diabetic patients (23). Biomarkers of AD and AD-related dementias (ADRD) play a crucial role in the accurate diagnosis of AD and ADRD. In longitudinal cohort of the Look AHEAD–Continuation study, which included overweight or obese elderly with T2D, increasing plasma levels of neurofilament light chain (NfL) and glial fibrillary acidic protein (GFAP) were found to be associated with impaired cognitive function, but Aβ_42/40_ or pTau-181 were not associated with cognitive decline (24).

## 3 Cause and risk factors linking T2D to cognitive impairment

**Figure 1:**
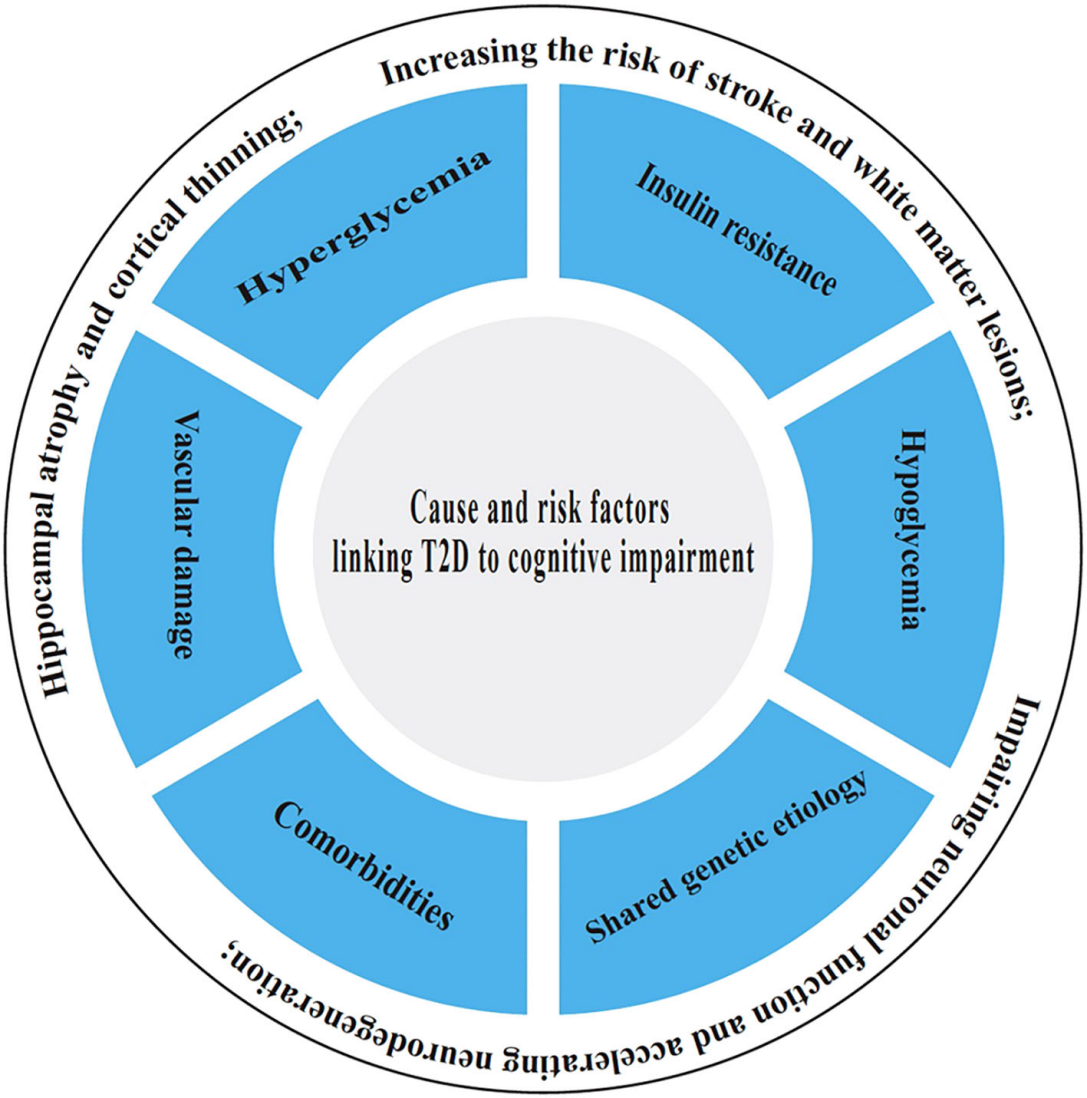
Causes and risk factors connecting T2D to cognitive impairment.

The underlying causes and risk factors linking T2D to cognitive impairment involve a complex interplay of metabolic disturbances, including chronic hyperglycemia and insulin resistance, which collectively lead to vascular damage, reduced cerebral blood flow, and microvascular complications, increasing the risk of stroke and white matter lesions (25). Additionally, prolonged high blood sugar levels promote oxidative stress, neuroinflammation, and the accumulation of advanced glycation end products (AGEs), which impair neuronal function and accelerate neurodegeneration. Other risk factors include obesity, hypertension, and dyslipidemia, which are common in T2D and further exacerbate cognitive decline (26). Poor glycemic control, longer diabetes duration, and recurrent hypoglycemic episodes also contribute to structural brain changes, such as hippocampal atrophy and cortical thinning. Genetic predisposition and lifestyle factors like physical inactivity and poor diet may further amplify the risk, highlighting the complex interplay between metabolic dysfunction and cognitive deterioration in T2D. It is as reflected by **Figure 1**.

### 3.1 Hyperglycemia

Chronically elevated blood glucose is a key factor linking T2D to MCI, dementia, and AD (27). Data from the China Health and Retirement Longitudinal Study (CHARLS) indicate that elevated long-term mean HbA1c levels are significantly linked to an increased risk of cognitive impairment (CI), particularly in the domains of global cognition and episodic memory (28). Prolonged exposure to high blood glucose levels induces oxidative stress, triggers inflammatory responses, and facilitates the formation of advanced glycation end products (AGEs), which cross-link proteins and lipids, thereby impairing neuronal function, exacerbating inflammation, and accelerating neurodegenerative processes (29). Persistent hyperglycemia also disrupts Blood-Brain Barrier (BBB) integrity, allowing harmful substances to enter the brain and trigger neuroinflammation (30). Chronic hyperglycemia in T2D damages small blood vessels, which reduces cerebral blood flow and leads to white matter hyperintensities, impairing connectivity between brain regions (31). Hyperglycemia may alter gene expression related to synaptic plasticity and neurodegeneration (32).

### 3.2 Insulin resistance

Insulin crosses the BBB and binds to receptors in neurons, supporting synaptic plasticity, memory formation, and glucose metabolism. Insulin resistance is a hallmark of T2D and plays a central role in cognitive decline (33). Insulin resistance in the brain reduces glucose uptake and energy deficits, and then neurons become energy-deprived due to impaired glucose utilization and synaptic plasticity (34). Neuronal insulin resistance disrupts long-term potentiation (LTP) which is critical for learning and memory, and decreases brain-derived neurotrophic factor (BDNF) resulting in neurodegeneration (35). Moreover, insulin-degrading enzyme (IDE) is the key molecular to clear both insulin and amyloid-β (Aβ). Hyperinsulinemia from insulin resistance competes for IDE, which may contribute to reduce Aβ clearance leading to increasing amyloid plaques (36). Insulin resistance also activates glycogen synthase kinase-3β (GSK-3β), promoting tau phosphorylation and neurofibrillary tangles (37). Furthermore, loss of insulin signal in microglia leads to mitochondrial dysfunction, diminishes the autophagy process, and promotes neuroinflammation. This cascade of events contributes to the accumulation of pro-inflammatory cytokines (such as IL-6, TNF-a), impaired Aβ clearance, and accelerates the progression of AD (38–40). A case-control study demonstrated that serum levels of IL-6 and high-sensitivity C-reactive protein (hs-CRP) were significantly associated with the risk of mild cognitive impairment (MCI) among Chinese patients with T2D (41). Additionally, chronic insulin resistance impairs endothelial function resulting in decreasing cerebral blood flow and breakdown of the BBB, allowing toxins and inflammatory molecules into the brain (42).

### 3.3 Vascular damage

Vascular damage is a critical pathway linking T2D to cognitive impairment and dementia. Chronic hyperglycemia and insulin resistance in T2D lead to endothelial dysfunction, BBB disruption, and micro- and macrovascular damage, impairing cerebral blood flow and promoting ischemia (43). Small vessel disease manifests as white matter hyperintensities, microinfarcts, and microbleeds, disrupting neural connectivity and accelerating cognitive decline (44). Additionally, diabetes exacerbates cerebral amyloid angiopathy (CAA) and atherosclerosis, increasing stroke risk and compounding neurodegeneration (45). Neuroimaging studies show that individuals with T2D exhibit greater white matter lesions and brain atrophy, correlating with poorer memory and executive function (46). Thus, vascular damage serves as a key mediator between T2D and dementia, highlighting the importance of early vascular risk factor management in diabetic patients.

### 3.4 Hypoglycemia

Severe or recurrent hypoglycemic episodes, often a side effect of diabetes treatment, contributes to cognitive impairment in T2D through multiple mechanisms (47). Data from the CHARLS cohort also suggest that excessively low HbA1c levels, as well as greater fluctuations in HbA1c, are associated with an increased risk of CI (28). Severe hypoglycemic episodes can lead to acute neuronal damage by depriving the brain of glucose, its primary energy source, resulting in synaptic dysfunction, oxidative stress, and even selective neuronal death, particularly in memory-related regions like the hippocampus (48). Recurrent hypoglycemia may also impair cognitive reserve over time, accelerating neurodegeneration and increasing dementia risk (49, 50). Additionally, hypoglycemia-induced inflammation and blood-brain barrier disruption further exacerbate cognitive decline. Epidemiological studies show that individuals with T2D who experience severe hypoglycemia have a higher risk of developing dementia, suggesting a bidirectional relationship between poor glycemic control and cognitive dysfunction (51–53). Therefore, balancing glycemic targets to avoid both hyper- and hypoglycemia is crucial for preserving brain health in diabetes management.

### 3.5 Comorbidities

Comorbidities of T2D significantly contribute to cognitive impairment through interconnected metabolic, vascular, and inflammatory pathways. Hypertension, commonly coexisting with T2D, exacerbates cerebrovascular damage by promoting small vessel disease, white matter lesions, and microinfarcts, which impair cognitive function (54, 55). Obesity and dyslipidemia drive systemic inflammation and insulin resistance, further compromising brain metabolism and increasing Aβ deposition (28, 56–59). Diabetic nephropathy reduces toxin clearance and promotes uremic encephalopathy, while peripheral neuropathy may limit physical activity, worsening cerebral blood flow (60). Sleep apnea, prevalent in T2D, induces chronic intermittent hypoxia and oxidative stress, accelerating hippocampal atrophy (61, 62). Depression, another frequent comorbidity, not only diminishes cognitive reserve but also shares underlying mechanisms with neurodegeneration, including hypothalamic-pituitary-adrenal axis dysfunction (63, 64). Several studies have suggested a causal relationship between depression and an increased risk of developing T2D, with both major depressive disorder and depressive symptoms showing a positive association with T2D. However, the causal relationship between depression and cognitive disorders in individuals with T2D remains to be further investigated (65–67). Insulin resistance plays an important role in the development of depressive symptom and cognitive decline in individuals with T2D (68). Together, these comorbidities create a synergistic assault on brain health, amplifying T2D’s direct neurotoxic effects and substantially elevating dementia risk. This highlights the importance of comprehensive, multi-system management in preserving cognitive function in diabetic patients.

### 3.6 Shared genetic etiology underlying AD and T2D

Emerging evidence reveals a shared genetic etiology between AD and T2D, suggesting common biological pathways drive both conditions (69). Genome-wide association studies (GWAS) have identified overlapping risk loci, including *APOE-ϵ4* (which influences lipid metabolism and amyloid clearance), *CLU* (involved in synaptic maintenance and glucose homeostasis), and *IDE* (insulin-degrading enzyme, which clears both insulin and amyloid-beta) (70, 71). Bioinformatics analysis of effective biomarkers in type 2 diabetes with cognitive impairment and aging indicates that the genes TP53 and IL1B may play a potential role in influencing the progression of type 2 diabetes associated with cognitive impairment and aging through the Lipid and atherosclerosis, MAPK signaling, and fluid shear stress and atherosclerosis signaling pathways (72). Polygenic risk scores for T2D correlate with higher AD incidence, while Mendelian randomization studies support a causal link between insulin resistance and neurodegeneration (73). Shared mechanisms include impaired insulin signaling in the brain, mitochondrial dysfunction, and chronic inflammation, which exacerbate amyloid and tau pathology. These findings highlight the role of metabolic dysregulation in AD pathogenesis and suggest that T2D and AD may represent different manifestations of a broader “metabolic-cognitive syndrome,” opening avenues for targeted therapies that address both conditions.

### 3.7 Gut microbiota and AD in T2D

Microbial balance plays a crucial role in maintaining glucose homeostasis and safeguarding cognitive function. Growing evidence has shown that an imbalance in gut microbiota is linked to the pathogenesis of T2D (74–76). Furthermore, it suggests that gut microbiota affects cognitive impairment associated with T2D via the gut-brain axis (77, 78). In the Hong study, it was reported that patients with diabetic cognitive dysfunction exhibited a reduced abundance of *Bifidobacterium* and *unnamed bacteria RF39*, along with an increased abundance of *Peptidococcus* and *Leucococcus (* 79). In the cognitive impairment db/db diabetic mouse model, fecal microbiota analysis revealed that species abundance and diversity in db/db mice were significantly higher compared to those in the control group (80). The microbiota-gut-brain axis includes neural, immune, endocrine, and metabolic pathways, but the communication system is not fully understood. Recent studies suggest that microbiota dysbiosis and T2D caused by long-term high-fat diet (HFD) increase permeability of the gut and blood-brain barrier mediated neurodegenerative disorders (81–83). In addition, diabetic cognitive impairment is associated with neuroinflammation induced by imbalance of the gut microbiota (84, 85).

## 4 Molecular mechanisms underlying cognitive impairment in T2D

The pathogenesis of cognitive decline in T2D is unclear and the exact molecular mechanisms are complex and multifactorial. Emerging research reveals that T2D-induced cognitive dysfunction involves intricate molecular pathways that disrupt neuronal homeostasis and synaptic plasticity (86). At the cellular level, chronic hyperglycemia activates the polyol pathway leading to advanced glycation end products (AGEs) formation. On the one hand, AGEs reduced the levels of O-Linked β-N-acetylglucosamine (O-GlcNAc) transferase (OGT), thereby downregulating O-GlcNAcylation and inducing tau protein hyperphosphorylation, which is implicated in diabetes-associated cognitive dysfunction (87). On the other hand, AGEs crosslink with the receptor of AGEs (RAGE), activating NF-κB and NLRP3 inflammasomes, perpetuating a vicious cycle of neuroinflammation (88). Moreover, this process impairs cerebral microvascular integrity and induces synaptic mitochondrial dysfunction (89, 90).

IDE is an atypical zinc-metalloprotease that plays a key role in regulating insulin and Aβ levels in the brain and peripheral tissues. It degrades both insulin and Aβ. Hyperinsulinemia refers to excess insulin levels in the blood due to insulin resistance. High insulin levels and insulin resistance are suggested to be associated with reduction of IDE (91). The cellular and molecular mechanisms underlying the relationship between hyperinsulinemia and IDE expression remain poorly understood. The novel insights into the regulation of IDE reveal that miR-7, miR-125, miR-490 and miR-199 downregulate IDE expression at the post-transcriptional level in response to high insulin levels. In addition, the authors found that IDE contains multiple potential binding sites for several RNA binding proteins (RBP) (92, 93). Furthermore, insulin receptor substrate-1 (IRS-1) proteins become less active due to the development of hyperinsulinemia, which arises from insulin resistance as a result of the reduced expression of insulin receptors (IR). The inactive of IRS1 leads to the down-regulation of the phosphoinositide 3-kinase (PI3K)/protein kinase B (Akt) pathway and the up-regulation of GSK-3β activity, and promotes tau hyperphosphorylation and Aβ accumulation in the brain (37, 94, 95).

The dentate gyrus (DG) of the hippocampus is a crucial brain region involved in memory encoding. Tang et al. found that neuronal ferroptosis in the hippocampus contributes to the initiation and development of learning impairment and memory processes in T2D mice (96, 97). Recent studies indicate that diabetes causes hippocampal neuronal damage and loss due to increased iron concentrations, MDA, and ROS levels, along with decreased GSH and GPX4. Furthermore, the underlying mechanism of neuronal ferroptosis is associated with the inhibition of Nrf2 in the hippocampus induced by T2D (88, 98). In recent years, a growing research interest in microbiota-gut-brain with T2D has been demonstrated that the gut microbiota plays an important role in the development of metabolic disorders. Specially, in the central nervous system (CNS), suggested by preclinical studies indicate that the restoration of intestinal microbiota may enhance cognitive function impaired by diabetes and ameliorate hippocampal neuron ferroptosis (77, 99).

In the latest study, authors identified that the miR-9-3p cargo in adipose tissue-derived extracellular vesicle (EVs) obtained from high-fat diet-fed mice or patients with T2D significantly suppressed BDNF levels in primary neurons, thereby inducing synaptic damage associated with obesity-related insulin resistance (33).

The large-scale proteomic analysis conducted as part of the UK Biobank Pharma Proteomics Project demonstrated that a 51-protein model exhibited excellent accuracy in predicting the 15-year risk of dementia among individuals with T2D. Furthermore, elevated levels of Rho guanine nucleotide exchange factor 12 (ARHGEF12) was specifically linked to an increased risk of dementia. Pathway analysis revealed that elevation of IL6-JAK-STAT3 signaling pathway was involved in the development of dementia in T2D patients. Additionally, dysregulation of fatty acid was identified as a specific mechanism associated with the pathogenesis of AD in the context of T2D (100). These results may have potential applications in early risk stratification and targeted interventions, and may indicate possible therapeutic targets.

**Figure 2:**
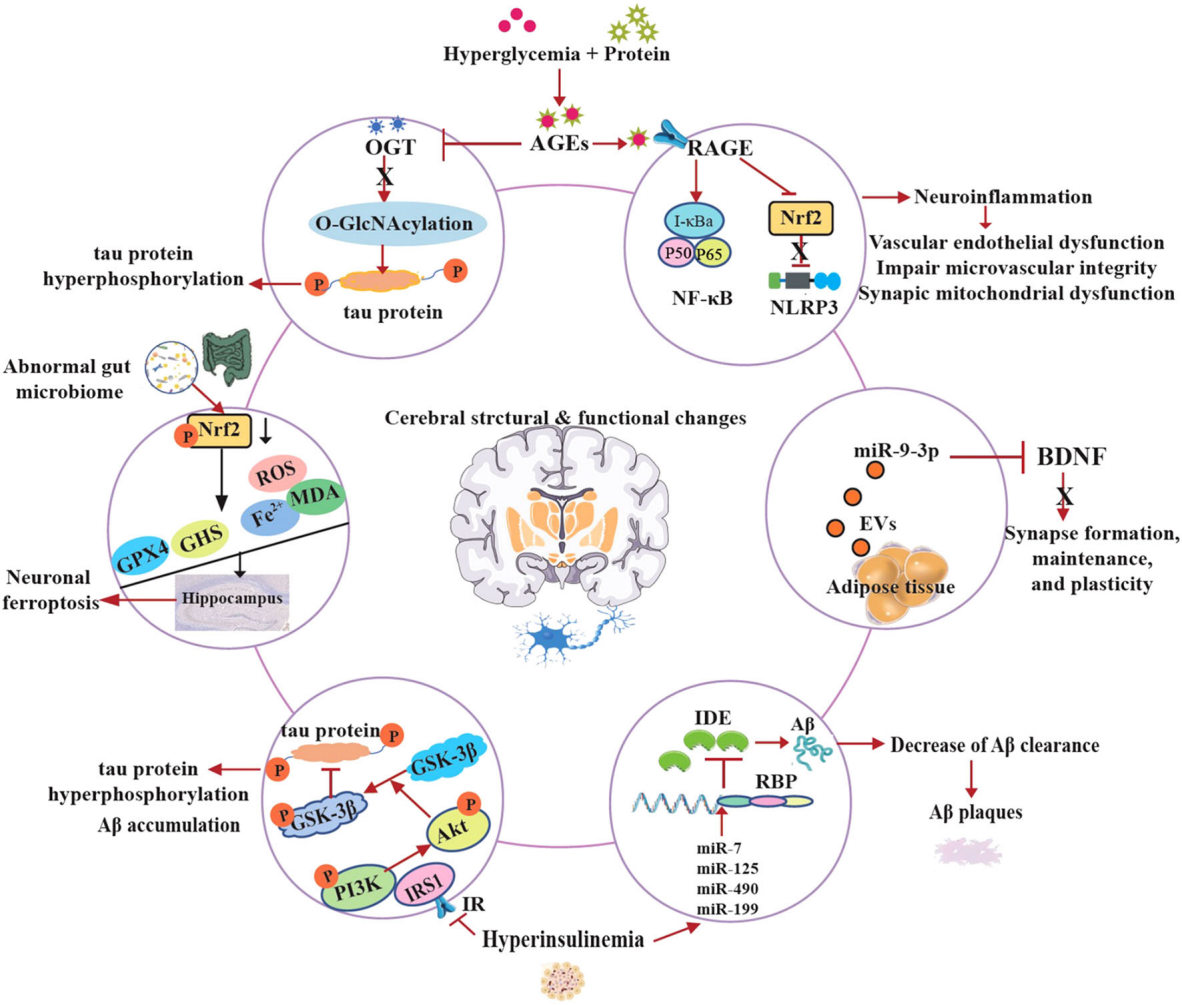
Molecular mechanisms contributing to cognitive impairment in T2D.

Overall, these evidences provide these molecular perturbations collectively drive synaptotoxicity, white matter degeneration, and accelerated amyloidogenesis, positioning T2D as a potent modifier of Alzheimer’s pathology (**Figure 2**). Therapeutic targeting of these pathways may offer neuroprotection in diabetic cognitive decline.

## 5 Evidence from intervention of cognitive impairment in T2D

**Figure 3:**
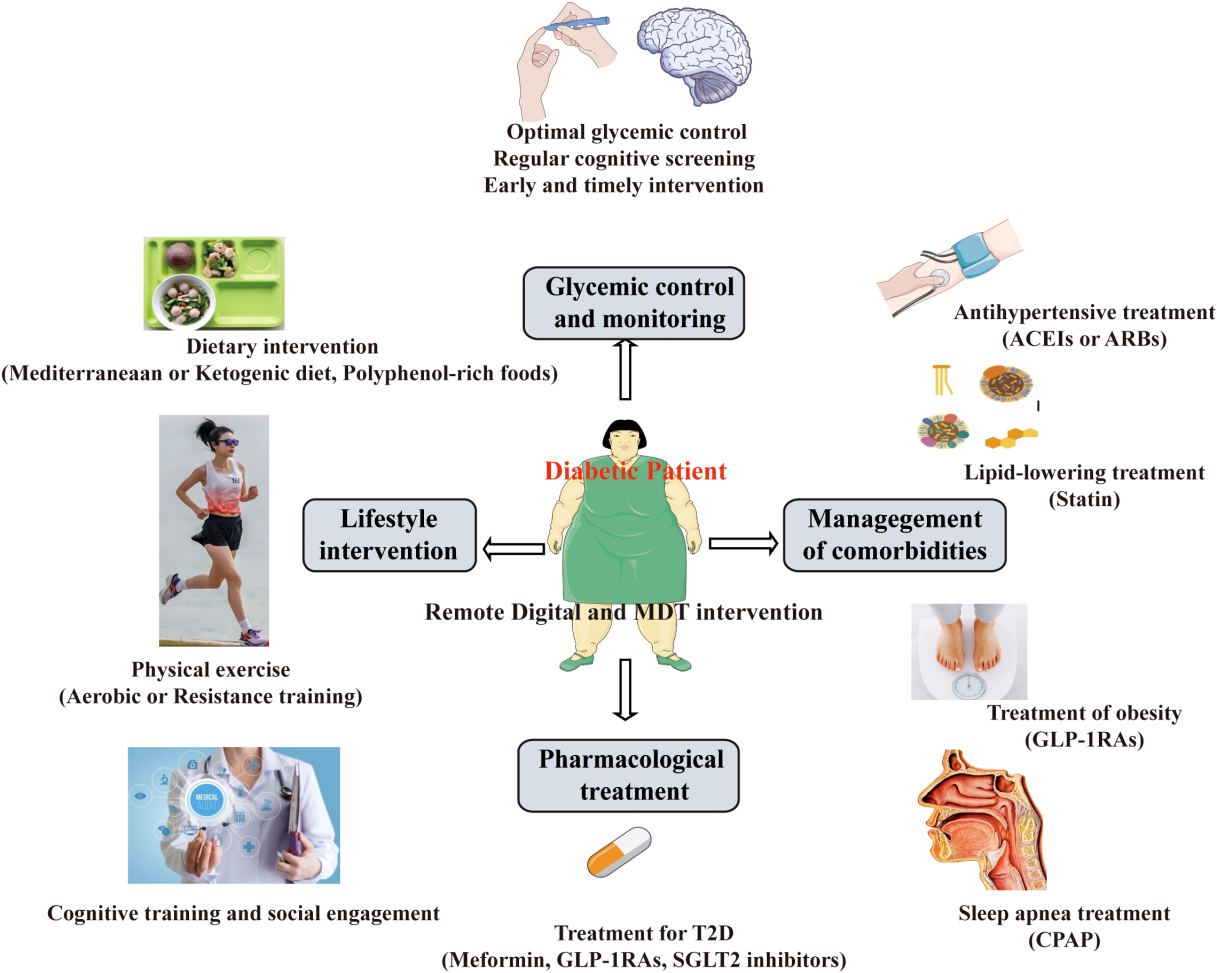
Intervention and management of cognitive impairment in T2D.

Cognitive dysfunction is often unrecognized in individuals with T2D. Cognitive dysfunction in T2D can have significant consequences on an individual’s overall health, quality of life, and disease management. Therefore, there is an urgent need to find effective therapeutic strategies to improve cognitive function among patients with T2D (**Figure 3**).

### 5.1 Glycemic control and monitoring

A growing body of clinical and experimental evidence demonstrates that optimal glycemic control may help prevent or delay cognitive decline in T2D, though the relationship is complex and influenced by treatment strategies (101–103). Longitudinal studies show a U-shaped association, where both hyperglycemia (HbA1c>8%) and recurrent severe hypoglycemia accelerate cognitive impairment (104, 105). The ACCORD MIND trial found that intensive glycemic control (HbA1c <6.0%) did not significantly improve cognition but increased hypoglycemia risk (106). Meta-analyses suggest HbA1c variability (fluctuations) is an independent predictor of dementia risk, possibly due to oxidative stress (107). The Look AHEAD trial (focused on weight loss + glycemic control) showed no significant cognitive benefit, possibly due to limited follow-up (108). The DCCT-EDIC study (Type 1 diabetes) found that tight glycemic control initiated early in the disease course has been associated with better long-term cognitive outcomes, where early intensive glucose management reduced later cognitive decline by 30-40% (109). Some RCTs suggest metformin may reduce dementia risk, while insulin therapy in older adults may worsen cognition if hypoglycemia occurs (110, 111).

Strong evidence indicates that proactive monitoring and early intervention in patients with T2D can significantly delay or mitigate cognitive impairment (112, 113). Longitudinal studies demonstrate that regular cognitive screening in diabetic populations enables earlier detection of subtle deficits, allowing for timely interventions before significant neurodegeneration occurs (114, 115).

The 2025 American Diabetes Association (ADA) clinical guidelines recommend that cognitive capacity should be monitored throughout the life span for all diabetic patients (116). The Mini-Mental State Examination (MMSE) and the Montreal Cognitive Assessment (MoCA) are the two most widely used assessing tools for cognitive function, and recommended by the guideline (117). Nonetheless, the MMSE and MoCA tests exhibit low sensitivity and specificity in detecting early stages of MCI. In recent years, retinal microperimetry has emerged as a valuable tool for monitoring cognitive function in diabetic patients (118, 119). Continuous glucose monitoring (CGM) technologies have proven particularly valuable, as they minimize glycemic variability - an independent risk factor for cerebral small vessel disease and cognitive dysfunction (120, 121). These findings underscore the importance of incorporating cognitive assessments into standard diabetes care protocols and implementing preventive strategies at the earliest detectable stage of metabolic dysfunction to optimally preserve brain health.

### 5.2 Lifestyle intervention

A robust body of research demonstrates that structured lifestyle interventions can significantly mitigate cognitive decline in individuals with T2D. These interventions primarily target diet, physical activity, weight management, and cognitive training, working through metabolic, vascular, and neuroprotective pathways (122, 123).

Emerging research highlights that targeted dietary interventions can significantly influence cognitive outcomes in individuals with T2D by modulating metabolic, vascular, and neurodegenerative pathways (124). Adherence to a Mediterranean diet, rich in polyphenols and omega-3 fatty acids, has been associated with improved memory and slower hippocampal atrophy, likely due to its anti-inflammatory and antioxidant properties (125, 126). Ketogenic diets and intermittent fasting enhance neuronal energy metabolism through ketone bodies and autophagy, improving cognitive performance in insulin-resistant individuals (78, 127, 128). Polyphenol-rich foods, such as berries and cocoa, further protect against neurodegeneration by modulating oxidative stress and neuroinflammation (129). Clinical trials, including PREDIMED and COSMOS, underscore the cognitive benefits of these dietary strategies, particularly in at-risk populations like APOE-ϵ4 carriers (130, 131). Together, these findings advocate for personalized, nutrient-dense dietary interventions as a viable approach to delay or prevent cognitive decline in T2D.

Emerging evidence demonstrates that physical exercise significantly attenuates cognitive impairment in T2D through multifaceted physiological mechanisms (132). Both aerobic and resistance training have been shown to improve memory, executive function, and processing speed in T2D patients, with neuroimaging studies revealing increased hippocampal volume and enhanced cerebral blood flow following regular exercise. Aerobic activities like brisk walking and cycling elevate brain-derived neurotrophic factor (BDNF) levels, promoting neurogenesis and synaptic plasticity, while resistance training reduces systemic inflammation and improves insulin sensitivity in brain tissue (133, 134). Combined exercise regimens appear particularly effective, as demonstrated in clinical trials such as Look AHEAD, where greater physical activity was associated with slower cognitive decline over time (108). Exercise also mitigates key pathological processes in T2D-related cognitive impairment, including reducing oxidative stress, improving cerebrovascular function, and decreasing Aβ accumulation (135, 136). In a Chinese randomized clinical trial found that Tai Chi Chuan improved global cognitive function more effectively than fitness walking in older adults with type 2 diabetes and MCI (137). These neuroprotective effects appear dose-dependent, with current guidelines recommending at least 150 minutes of moderate-intensity exercise weekly for optimal cognitive benefits (138). The findings underscore structured physical activity as a potent, non-pharmacological intervention to preserve brain health in diabetic populations.

Growing evidence suggests that cognitive training and social engagement interventions can help mitigate cognitive impairment in individuals with T2D by enhancing neural resilience and compensatory mechanisms (139). Computerized cognitive training programs targeting memory, attention, and executive function have demonstrated efficacy in improving processing speed and working memory in T2D patients, with neuroimaging studies showing increased functional connectivity in prefrontal and parietal regions (140, 141). Social engagement, including group activities and interactive cognitive stimulation, appears to provide additional benefits by reducing stress-related cortisol exposure while promoting cognitive reserve through complex social interactions (142). The Finnish Geriatric Intervention Study (FINGER) demonstrated that multidomain interventions combining cognitive training, social engagement, and lifestyle modifications significantly reduced dementia risk in at-risk populations, including those with metabolic disorders (143, 144). These approaches may be particularly valuable for T2D patients as they address both the direct neurological consequences of hyperglycemia and the psychosocial factors that often accompany chronic disease. While optimal protocols remain under investigation, current evidence supports incorporating structured cognitive exercises and social activities into comprehensive care plans for diabetes-related cognitive protection.

### 5.3 Pharmacological treatment

Emerging evidence suggests that certain pharmacological treatments for T2D may also help prevent or mitigate cognitive impairment, though findings remain nuanced. Metformin, the first-line antidiabetic medication, has demonstrated neuroprotective properties in observational studies, with some evidence linking its use to reduced dementia risk, potentially through AMPK activation and reduced neuroinflammation (145, 146). A case-control study of patients with type 2 diabetes (T2D) from the Memory Clinic at Hebei General Hospital indicates that long-term use of metformin is associated with reduced rates of cognitive impairment and a decreased burden of cerebral small vessel disease among patients with T2D (147). Data analysis from the global AD Neuroimaging Initiative (ADNI) study indicated that metformin treatment in T2D patients was associated with a positive effect on cognitive performance (148).

GLP-1 receptor agonists (GLP-1RAs, e.g., liraglutide, semaglutide) show promising candidates for dual glycemic and cognitive management, with preclinical studies demonstrating their ability to reduce Aβ accumulation, enhance synaptic plasticity, BDNF modulation, and improve cerebral blood flow (77, 149, 150). While not currently approved for cognitive outcomes in T2D, in a *post hoc* analysis of three cardiovascular outcome trials (LEADER, SUSTAIN 6, and PIONEER 6), patients with GLP-1RAs treatment represented a statistically significant 53% lower risk of all-cause dementia diagnosis compared to patients with placebo (149). In an exploratory analysis of the REWIND trail, patients with long-term treatment with the GLP-1RA dulaglutide also demonstrated reduction of cognitive decline (151). In the ELAD study, patients on liraglutide maintained greater temporal lobe and total cortical volume compared to those on placebo, along with better cognitive function preservation (152). The ongoing evoke and evoke+ are the first trials investigating the efficacy, safety, and tolerability of oral semaglutide in early-stage symptomatic AD (153). If both trails are successful, semaglutide may be considered for future treatment of AD. Conversely, insulin therapy in elderly patients with T2D requires careful consideration, as it may increase hypoglycemia-related cognitive risks.

SGLT2 inhibitors are medications used to manage T2D by preventing glucose absorption in renal tubules. They may provide cerebrovascular protection by reducing oxidative stress and improving endothelial function, although direct cognitive benefits remain under investigation (154, 155). The latest evidence demonstrates that SGLT2 inhibitors may offer cognitive benefits in patients with T2D through multiple protective mechanisms (12, 154, 156). Clinical observational studies report a 10-30% lower incidence of dementia among SGLT2 inhibitor users compared to other antidiabetic medications, with particular benefits seen for vascular cognitive impairment (157–159). The EMPA-REG OUTCOME trial’s subanalysis found empagliflozin-treated patients had slower progression of cognitive decline, potentially linked to its hemodynamic effects and ketone-mediated neuroprotection (160). While dedicated cognitive endpoint trials are ongoing, current evidence positions SGLT2 inhibitors as promising dual-purpose agents for both glycemic control and potential cerebrovascular protection in T2D, though their precise neurocognitive effects require further validation through randomized controlled trials with comprehensive cognitive assessments.

SGLT2 inhibitors may exert cognitive benefits in T2D through multiple interconnected molecular mechanisms. By inducing mild ketosis, these agents provide alternative cerebral energy substrates (β-hydroxybutyrate) that bypass insulin-resistant glucose metabolism, supporting neuronal function during metabolic stress (161). Their systemic metabolic effects including activation of AMPK, enhanced cerebral ketone metabolism, a shift in microglial activation from the pro-inflammatory phenotype to the anti-inflammatory phenotype, and reduced oxidative stress, contribute to the mitigation of neuroinflammation through the suppression of the NLRP3 inflammasome and AGE-RAGE signaling pathways (162, 163). At the cellular level, SGLT2 inhibitors may exert neuroprotective effects through increasing brain-derived neurotrophic factor (BDNF) expression, ameliorating mitochondrial dysfunction, while inhibiting-mediated tau phosphorylation (pTau) (164–167). Emerging evidence also suggests gut microbiome modulation, with increased production of neuroprotective short-chain fatty acids (168, 169). Additionally, SGLT2 inhibitors attenuated pTau accumulation by modulating brain insulin signaling through the angiotensin-converting enzyme 2/angiotensin (1–7)/mitochondrial assembly receptor axis in a T2D-AD mouse model (170). These pleiotropic effects position SGLT2 inhibitors as promising multitarget therapeutic agents for addressing diabetes-related cognitive impairment; however, additional clinical validation is required to confirm their efficacy and safety.

### 5.4 Management of comorbidities

The bulk of the evidence proves that comprehensive management of T2D comorbidities significantly improves cognitive outcomes by addressing multiple interconnected pathological pathways. Tight glycemic control and concurrent management of hypertension with ACE inhibitors or ARBs preserves cerebrovascular integrity, while statin therapy may mitigate both vascular cognitive impairment and neurodegenerative pathology through pleiotropic effects (171–173). Treatment of obesity with lifestyle interventions or GLP-1RAs not only improves metabolic parameters but also enhances neurogenesis and reduces neuroinflammation (174, 175). Additionally, addressing sleep apnea with CPAP therapy improves cerebral oxygenation, and antidepressant treatment for comorbid depression helps restore neurotrophic factor signaling (176). Multidomain interventions that simultaneously target glycemic control, vascular risk factors, and lifestyle modifications - as demonstrated in trials like the FINGER study - show particularly robust cognitive benefits, suggesting that a holistic approach to T2D management may be more effective than isolated therapies for preserving brain health in diabetic patients (177).

### 5.5 Remote digital technologies for interventions

Remote digital technologies, often referred to as “Digital Health” or “eHealth”, provide scalable, accessible, and cost-effective solution for addressing cognitive decline in patients with diabetes (178). These technologies utilize smartphones, tablets, wearable devices, and web-based platforms to provide cognitive assessment, training, monitoring, and comprehensive interventions directly to patients in their home environments (179–184). Remote digital technology for diabetic cognitive care intervenes at multiple levels.

To facilitate the early identification of subtle cognitive changes and to monitor their progression over time without the necessity for frequent, in-person neuropsychological assessments, Computerized Cognitive Tests and Digital Biomarkers are employed to evaluate and track cognitive alterations (185, 186). Computerized Cognitive Tests are validated, game-like assessments conducted on tablets or computers that evaluate memory, attention, executive function, and processing speed (187). These tests often demonstrate greater sensitivity to change compared to traditional paper-and-pencil assessments (188). Digital biomarkers assess cognitive states by utilizing passive data gathered from smartphones and wearable devices (189). This includes metrics such as keystroke dynamics, voice analysis, and gait analysis.

To enhance or maintain cognitive function, structured and repetitive exercises that promote neuroplasticity are utilized. This includes Brain Training Apps, Serious Games (Gamification), and Virtual Reality (VR) for cognitive training and rehabilitation (Cognitive Therapeutics) (190, 191). Brain training apps like BrainHQ, CogniFit, and Lumosity provide games for specific cognitive areas that can be prescribed and monitored remotely by clinicians (192–194). Serious Games (Gamification) enhance patient engagement, leading to better adherence to cognitive exercises.

To provide human support, guidance, and accountability, video conferencing, secure messaging platforms, and remote patient monitoring (RPM) platforms are integrated into telehealth and remote coaching care planning (195, 196). Patients can have virtual consultations with endocrinologists, neurologists, diabetes educators, or neuropsychologists via video conferencing. Secure Messaging Platforms provide asynchronous communication for patients and clinician (197). Clinicians aggregate data from CGMs, wearables, and cognitive apps via RPM platforms, enabling care teams to monitor a patient’s overall health and intervene proactively.

In summary, Remote digital technologies signify a paradigm shift in the management of diabetes-associated cognitive decline. These technologies facilitate a transition from a reactive, clinic-centric approach to a proactive, continuous, and patient-centered care model. By integrating cognitive training, glycemic monitoring, and lifestyle coaching into a unified remote platform, we gain a powerful means to not only enhance diabetes management but also safeguard brain health and preserve patients’ quality of life. The next step involves validating these tools and embedding them seamlessly into standard clinical practice.

### 5.6 A multidisciplinary team intervention

Diabetes-associated cognitive decline results from a complex interplay of metabolic, vascular, inflammatory, and comorbid factors. Therefore, no single healthcare professional can effectively address all these domains. A multidisciplinary team (MDT) intervention and collaborative care model are crucial components in effectively addressing the issue (198–200). An effective MDT for this population includes an endocrinologist, a neurologist, a certified diabetes educator, a registered dietitian nutritionist, a pharmacist, a psychologist, a care manager, a physical therapist, and a family member, all working within an integrated system of care. An endocrinologist initiates annual cognitive screening (using tools like MoCA or Mini-Cog) in high-risk T2D patients. When abnormal results are identified, a referral to neurology for formal diagnostic evaluation is initiated, and the rest of care team is alerted. The MDT then convenes to review the patient’s case and develops a unified, patient-centered care plan. The nurse care manager routinely monitors patient outcomes, such as HbA1c levels and hypoglycemic events, through a shared electronic health record system and conducts follow-ups to ensure the effectiveness of the care plan. The social worker and psychologist actively collaborate with caregivers, offering comprehensive training, essential resources, and emotional support to enhance their capacity in providing care.

In summary, managing cognitive impairment in T2D exemplifies the success of collaborative care. An MDT model effectively addresses the biological, psychological, and social complexities of the disease, breaking the cycle and enabling patients to live safer, higher-quality lives. It represents the standard that healthcare systems should aim for.

## 6 Conclusion

Cognitive impairment in T2D is driven by multiple interrelated mechanisms, including chronic hyperglycemia, insulin resistance, neuroinflammation, oxidative stress, and vascular dysfunction, which collectively contribute to neurodegeneration and cognitive decline. Addressing these impairments requires a comprehensive management strategy that combines optimal glycemic control (prioritizing medications with potential neuroprotective benefits, such as GLP-1RAs and SGLT2 inhibitors), lifestyle modifications (e.g., aerobic exercise, Mediterranean diet, and cognitive training), and aggressive management of cardiovascular risk factors (hypertension, dyslipidemia). Emerging therapies targeting neuroinflammation, mitochondrial dysfunction, and insulin signaling in the brain hold promise but require further clinical validation. Artificial intelligence (AI) will be utilized to personalize cognitive training programs, predict cognitive decline through digital biomarkers, and deliver adaptive coaching. Socially Assistive Robots will provide companionship to older adults with more advanced impairments and remind them to engage in cognitive exercises or take their medication. Future research should focus on identifying early biomarkers, developing personalized interventions, and conducting long-term trials to establish evidence-based approaches for preventing and treating diabetes-related cognitive decline. A proactive, multidisciplinary approach is essential to mitigate cognitive deterioration and improve quality of life in T2D patients.
